# Cigarette smoke promotes COPD by activating platelet-activating factor receptor and inducing neutrophil autophagic death in mice

**DOI:** 10.18632/oncotarget.20353

**Published:** 2017-08-18

**Authors:** Xiao-Xi Lv, Shan-Shan Liu, Ke Li, Bing Cui, Chang Liu, Zhuo-Wei Hu

**Affiliations:** ^1^ Immunology and Cancer Pharmacology Group, State Key Laboratory of Bioactive Substance and Function of Natural Medicines, Institute of Materia Medica, Chinese Academy of Medical Sciences & Peking Union Medical College, Beijing, 100050, P.R. China; ^2^ Institute of Medicinal Biotechnology, Chinese Academy of Medical Sciences & Peking Union Medical College, Beijing, 100050, P.R. China

**Keywords:** autophagy, elastase, emphysema, inflammation, PAF

## Abstract

Neutrophils are the most important effector cells during the development of chronic obstructive pulmonary disease (COPD). Although neutrophil elastase is critical in cigarette smoke (CS)-induced lung parenchyma, the mechanism by which CS triggers elastase release from neutrophils remains unclear. Here we report that CS induction of autophagy in neutrophils by activating platelet- activating factor receptor (PAFR) promotes COPD progression in mouse. We found that the dead neutrophils were increased in bronchoalveolar lavage fluid from CS-exposed mice. Blocking PAFR suppressed the CS-induced autophagy in neutrophils, protected neutrophils from death, and reduced elastase release. Mechanistically, CS enhanced ROS production and High mobility group box 1 (HMGB1) expression through activation of PAFR. The elevated HMGB1 interacted with beclin1, which promoted the dissociation of Bcl-2 from beclin1 and the assembly of autophagy core complex. Moreover, the antagonism of PAFR by rupatadine, a prescription PAFR inhibitor, protected against the development of emphysema, and reduced the autophagic death of neutrophils after CS exposure. These results suggest that CS contributes to the pathogenesis of COPD partly by inducing a PAFR-dependent autophagic death of neutrophils. Therefore, PAFR may be a therapeutic target for COPD and inhibition of PAFR may provide potential therapeutic benefits in the treatment of patients with COPD.

## INTRODUCTION

Chronic obstructive pulmonary disease (COPD) is one of the most common respiratory diseases that threaten the lives of the patients [1]. The most classic pathologic features of COPD are pulmonary emphysema caused by the irreversible destruction of the alveoli and airway obstructions caused by airway inflammation and remodeling (e.g. airway fibrosis) [2]. Generally, persistent and repeated injury of the alveolar epithelium and inflammation caused by inhaled insults such as cigarette smoke (CS) are the most important driving forces for the development of COPD [3]. Moreover, the immune microenvironment of the lung tissue which is maintained by neutrophils, macrophages, lymphocytes, epithelial cells and fibroblasts also promotes the progression of this disease [4, 5]. Indeed, several studies demonstrated that the numbers of immune cells are increased in the airway lumen in patients with COPD [5]. CS and other inhaled insults in the airway stimulate resident macrophages and epithelial cells to release large quantity of chemokines, which recruit neutrophils, monocytes and lymphocytes into the damaged tissue [6, 7]. Neutrophils are the key cells in innate immune system which can remove pathogens through phagocytosis and degranulation. An increase in airway neutrophil recruitment can be observed during COPD exacerbation, and the amount of neutrophils correlate with the decline of lung function [8]. In the lung, neutrophils secrete elastase, cathepsin G and proteinase-3 followed by cigarette smoke stimulation, which contribute to alveolar destruction [9]. In addition, a significantly increased number of apoptotic neutrophils have been found in sputum from COPD patients [10], and these dead neutrophils are important source of neutrophil elastase (NE). On the other hand, CS induces inflammation and oxidative stress through activating various receptors on cells. Platelet-activating factor receptor (PAFR) is a G-protein-coupled receptor that may be involved in the pathogenesis of COPD. It has been reported that the expression of PAFR is increased in airway epithelial cells from COPD patients [11, 12], and that CS exposure can promote the production of platelet-activating factor (PAF) [13]. In addition, PAF can facilitate the adhesive function of neutrophils, and has the potential to promote neutrophil recruitment [14, 15]. Nevertheless, whether neutrophil participates in CS-induced COPD through PAFR remains unknown.

Autophagy is a conserved lysosomal degradation process in eukaryotic cells, which can be activated by many stimuli, including pathogen-associated molecular patterns (PAMPs), danger-associated molecular patterns (DAMPs) and endoplasmic reticulum (ER) stress [16]. Basal autophagy is required for the clearance of unfolded or misfolded proteins, macromolecules and damaged organelles in injured cells; however, overactive autophagic flux could trigger autophagic cell death [17]. Several studies have indicated the role of autophagy activity in inflammatory pulmonary diseases like acute lung injury and pulmonary fibrosis [18–20]. It has also reported that autophagic proteins are increased in lung tissue from COPD patients [21]; and cigarette smoke extract (CSE) can induce autophagy in airway epithelial cells [22], which facilitates oxidative stress and DNA damage [23]. However, the biological link between autophagy and neutrophils activity in the pathogenesis of COPD and if CS can induce autophagy in neutrophils remain elusive.

In this study, we proposed that PAFR participates in CS-induced autophagy in neutrophils during the development of COPD. We found that CSE-induced neutrophils death and NE releasing depend partly on the activation of PAFR. Activation of PAFR in response to CSE exposure enhances the expression of HMGB1 by inducing reactive oxygen species (ROS) generation in neutrophils, which decreases the association of Bcl-2 and Beclin-1, promotes autophagic flux and autophagic cell death. Pharmacologic inhibition of PAFR by rupatadine, a prescription PAFR antagonist, protects against the development of the CS induced COPD in mouse. Collectively, these findings not only reveal a new mechanism of COPD development, but also suggest that PAFR on neutrophils is a promising target for COPD therapy.

## RESULTS

### The expression of PAFR is increased in neutrophils from cigarette smoke-induced COPD mice

**Figure 1:**
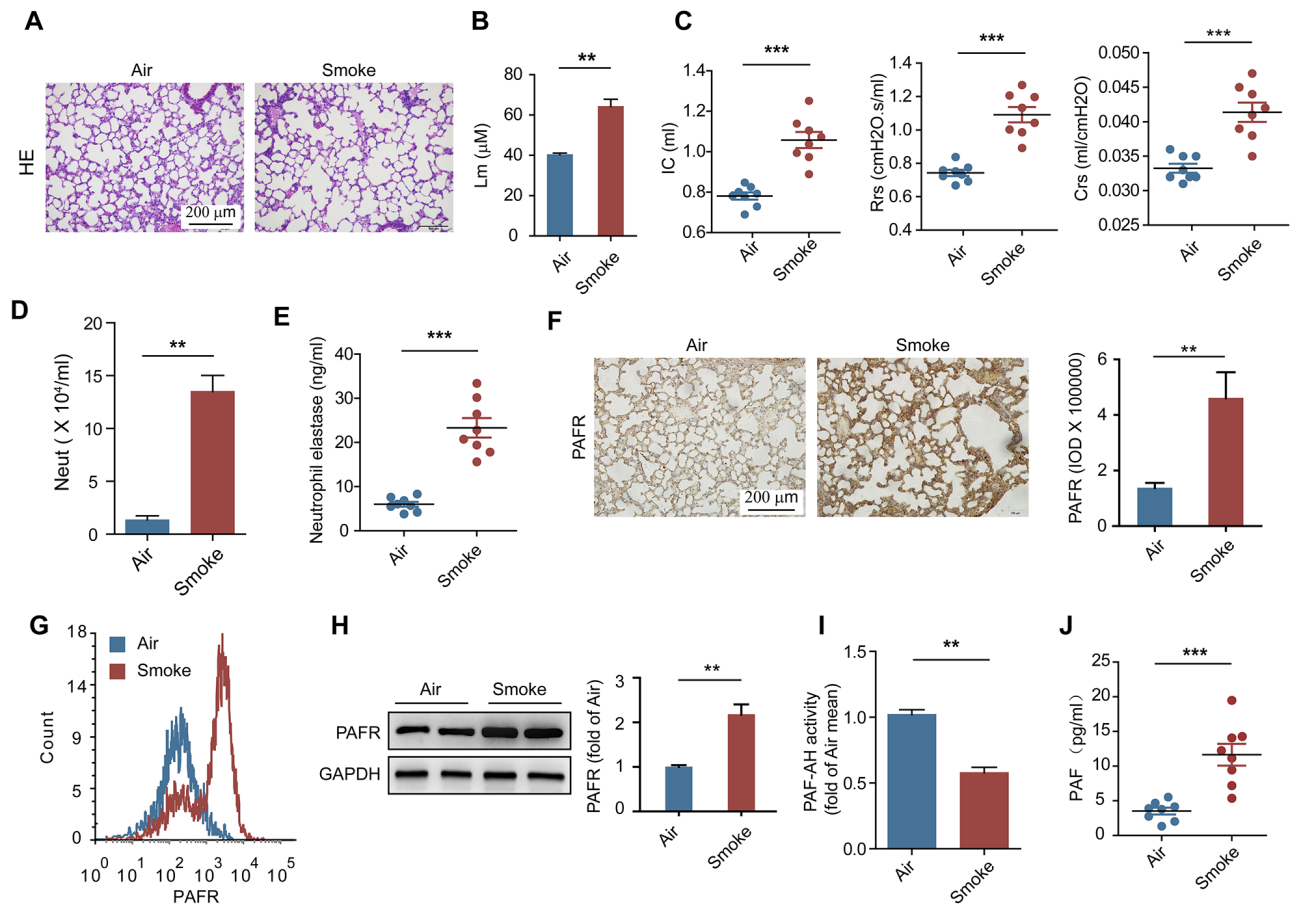
PAFR is involved in the pathogenesis of CS induced COPD **(A-E)** H&E-staining (A), the mean linear intercept of lung tissue (B), lung function (inspiratory capacity, dynamic resistance and dynamic compliance) (C), the number of neutrophils in BALF (D), and the concentration of neutrophil elastase in BALF (E) were detected to evaluate the development of COPD in mice after air or CS exposure for 16 weeks (n = 8). **(F)** Quantitative histopathology analyses of PAFR expression in lung samples from control and COPD mice (n = 8). **(G** and **H)** The expression of PAFR on neutrophil in BALF from CS exposed mice was detected with flow cytometry (G) and Western blot (H) (n = 6). **(I)** The activity of PAF-AH in BALF from the indicated mice was evaluated with PAF Acetylhydrolase Activity Assay Kit (n = 8). **(J)** The concentration of PAF was analyzed with ELISA in BALF from the indicated mice (n = 8). Data are mean ± SEM. Statistical significance between two groups was determined by student t test. *P < 0.05, **P < 0.01, ***P < 0.001.

A 16-week chronic model of CS exposure was used to mimic COPD in C57BL/6 mice [24–26]. Histopathological examination revealed that the alveolar space were enlarged (Figure 1A) and the mean linear intercept were increased (Figure 1B) after CS exposure. Furthermore, the increases in inspiratory capacity, lung resistance and lung compliance were observed in CS-exposed mice as compared to control mice (Figure 1C), indicating a decline of the lung function in these mice. CS exposure was associated with the increased number of neutrophils and the quantity of NE in bronchoalveolar lavage fluid (BALF) from CS-exposed mice (Figure 1D and 1E), suggesting that smoke stimulation induces the lung injury and COPD accompanied with the activation of neutrophils. Previous studies showed that platelet-activating factor (PAF) can induce neutrophil adhesion and activation by cooperation with selectin signaling [27, 28]. Compared with control mice, the expression of PAFR was enhanced in lung tissue and on neutrophils in BALF from CS- exposed mice (Figure 1F-1H). Moreover, higher concentration of PAF and lower activity of platelet-activating factor acetylhydrolase (PAF-AH) were detected in BALF from mice after chronic CS stimulation (Figure 1I and 1J), which may format a feedback control mechanism to keep the PAFR signaling pathway activation [13]. These results suggest that activation of PAFR signaling in neutrophils is involved in the pathogenesis of CS-induced COPD.

### CSE-induced autophagy is partly dependent on PAFR in neutrophils

**Figure 2:**
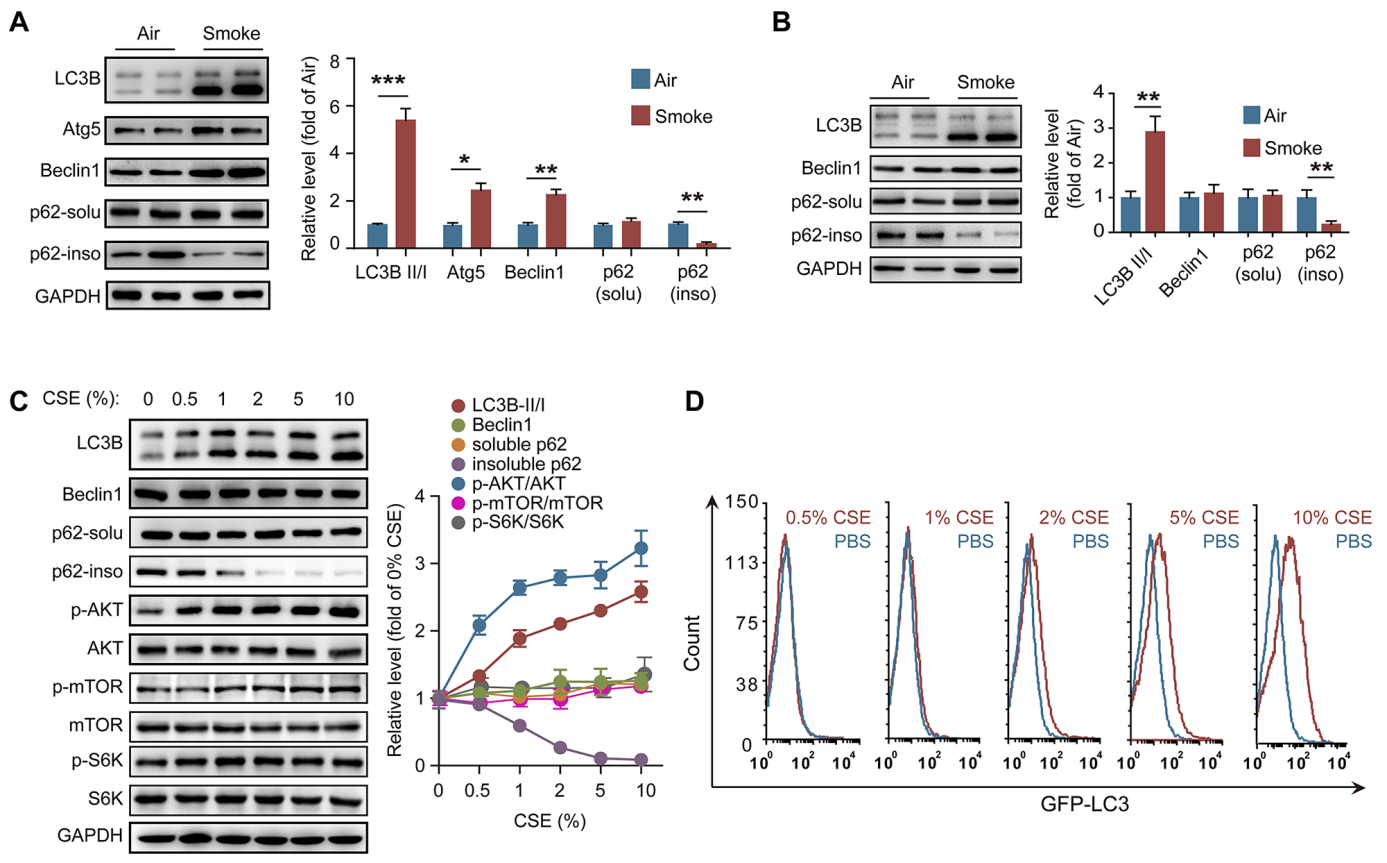
CS activates autophagy in neutrophils **(A)** The expression of autophagy related proteins in lung tissues were evaluated with Western blot. Data are representative immunoblots and the ratios of the indicated protein to GAPDH. (n = 6). **(B)** The level of autophagy related proteins in neutrophils from BALF were evaluated with Western blot (n = 6). **(C)** Neutrophils from bone marrow (BM) were treated with different concentration of CSE for 24 hr, and the proteins level was determined with Western blot. Data are representative immunoblots and the ratios of the indicated protein to GAPDH (n = 3). **(D)** BM neutrophils overexpressed GFP-LC3 were treated with different concentration of CSE for 24 hr, and the GFP fluorescent in cells was detected with flow cytometer after washed with 0.05% saponin (n = 3). Data are mean ± SEM. Statistical significance between two groups was determined by student t test; *P < 0.05, **P < 0.01, ***P < 0.001.

**Figure 3:**
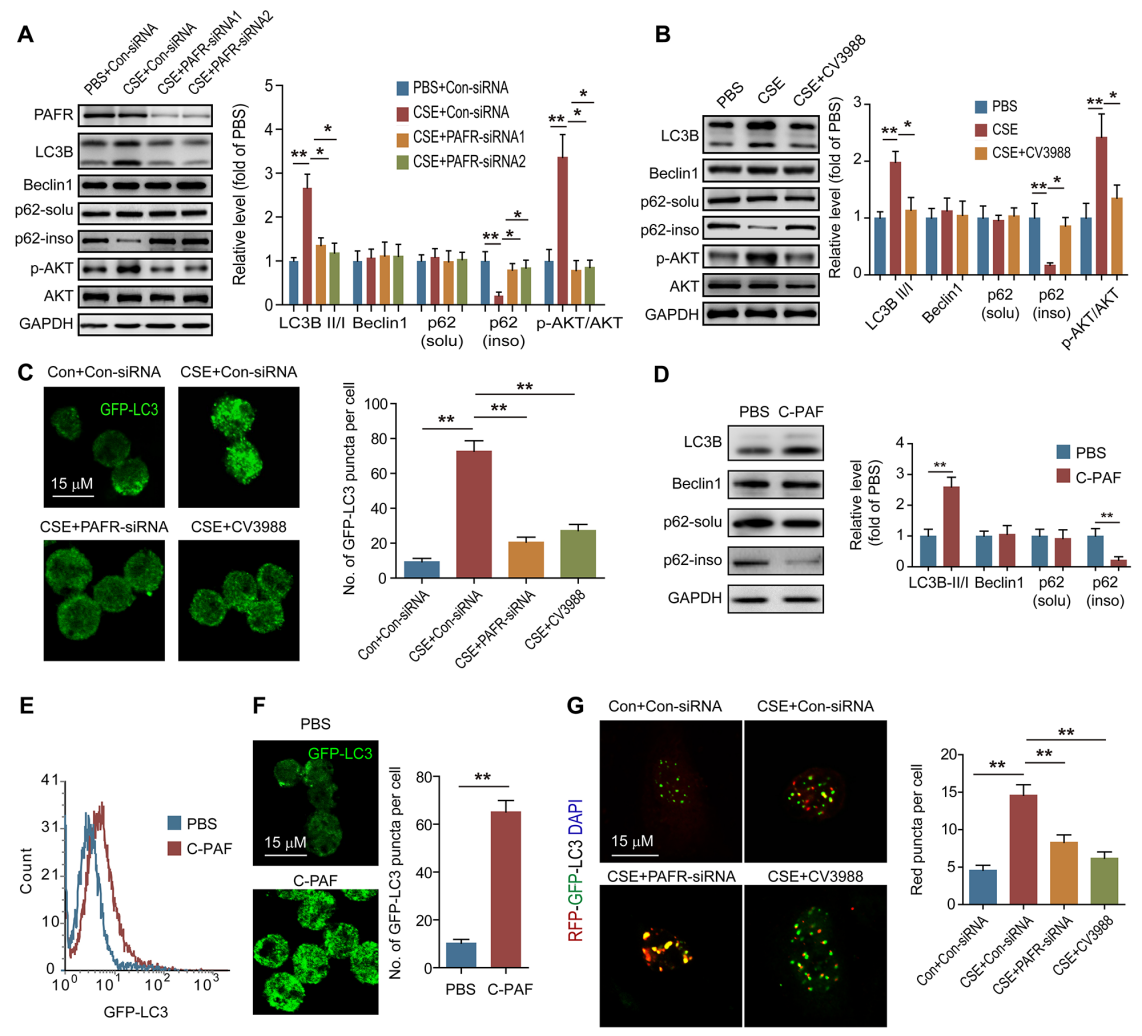
PAFR mediates CS-induced autophagy in neutrophils **(A)** PAFR silenced BM neutrophils were stimulated with 5% CSE for 24 hr, and the protein levels were examined with Western blot. Data are representative immunoblots and the ratios of the indicated protein to GAPDH (n = 3). **(B)** The expression of autophagy related proteins in BM neutrophils were determined with Western blot after treatment with 5% CSE or CSE plus CV3988 (30 μM) for 24 hr (n = 3). **(C)** The LC3 puncta in BM neutrophils overexpressing GFP-LC3 were evaluated with confocal after indicated treatment (n = 3). **(D)** BM neutrophils were treated with C-PAF (5 μg/ml) for 24 hr, and autophagy related proteins were analyzed with Western blot. Data are representative immunoblots and the ratios of the indicated protein to GAPDH. (n = 3). **(E)** GFP-LC3 overexpressed BM neutrophils were treated with C-PAF (5 μg/ml) for 24 hr, and the GFP fluorescent in cells was detected with flow cytometer after washed with 0.05% saponin (n = 3). **(F)** The LC3 puncta in GFP-LC3 overexpressed BM neutrophils were evaluated with confocal after stimulated with 5 μg/ml of PAF for 24 hr (n = 3). **(G)** The fluorescent of BM neutrophils infected with mRFP-GFP-LC3 adenovirus were analyzed with confocal after indicated treatments (n = 3). Data are mean ± SEM. Statistical significance between two groups was determined by student t test; statistical significances among groups were determined by one-way ANOVA test; *P < 0.05, **P < 0.01, ***P < 0.001.

It has been confirmed that CS can induce autophagy in lung epithelial cells, fibroblasts and macrophages [22, 29]. We found that the ratios of LC3B-II/I level, as well as the expression of Atg5 and beclin1, were increased in lung tissue from COPD mice (Figure 2A). As a marker of autophagic flux, p62 is involved in the degradation of unfolded or misfolded proteins in cells, and the content of insoluble p62 is an indicator of autophagy activation [30]. We observed that the amount of insoluble p62 but not soluble p62 was significantly decreased in lung tissue after CS exposure, suggesting that CS activates autophagy in lung tissue (Figure 2A). We then isolated the neutrophils in BALF from control and COPD mice to observe the autophagy activity in these cells. Interestingly, inconsistent with the increment of LC3B-II level and reduction of insoluble p62 amount, the beclin1 level in neutrophils from BALF was not changed after CS exposure (Figure 2B), indicating that CS-induced autophagy in neutrophils is independent of enhancing beclin1 expression. To determine the mechanism of CS-induced autophagy in neutrophils, neutrophils were isolated from bone marrow (BM) of C57BL/6 mice, and treated with different concentrations of CSE. CSE stimulation increased the ratio of LC3B-II/I, and reduced the amount of insoluble p62 in a concentration-dependent manner, but the beclin1 expression was not enhanced after CSE treatment (Figure 2C). As the cellular energy metabolism signals, the PI3K/AKT/mTOR pathway negatively regulates the activity of autophagy [31]. However, CSE significantly increased the ratio of p-AKT/AKT in neutrophils without changing the ratio of p-mTOR/mTOR and p-S6K/S6K, which suggests that CSE triggered autophagy is not dependent on activation of this upstream signaling (Figure 2C). Using GFP-LC3 plasmid, we found that CSE promoted the formation of autophagosome in neutrophils (Figure 2D). Knockdown PAFR or treatment of the cells with PAFR inhibitor CV3988 suppressed the CSE-induced LC3B-I to LC3B-II conversion, reduced the phosphorylation of AKT, and increased the insoluble p62 expression (Figure 3A and 3B). Using immunofluorescence technology, we found that silencing or inhibiting PAFR decreased LC3 fluorescent punctas in autophagosomes in neutrophils after CSE stimulation (Figure 3C). Moreover, activation of PAFR with C-PAF induced autophagy in neutrophils as indicated by the reduction of insoluble p62 content and the formation of autophagosome (Figure 3D-3F). To further verify whether CSE could induce autophagic flux in neutrophils, the flux rate of autophagy was evaluated using mRFP-GFP-LC3 adenovirus [32]. CSE treatment increased red punctas in neutrophils, suggesting CSE enhances autophagic flux in these cells. In contrast, silencing or inhibiting PAFR blocked CSE-induced autophagy as indicated by an increase in yellow punctas in neutrophils (Figure 3G). These data indicate that PAFR mediates CS-induced autophagy in neutrophils.

### PAFR mediates CSE induced neutrophil death via inducing autophagy

**Figure 4:**
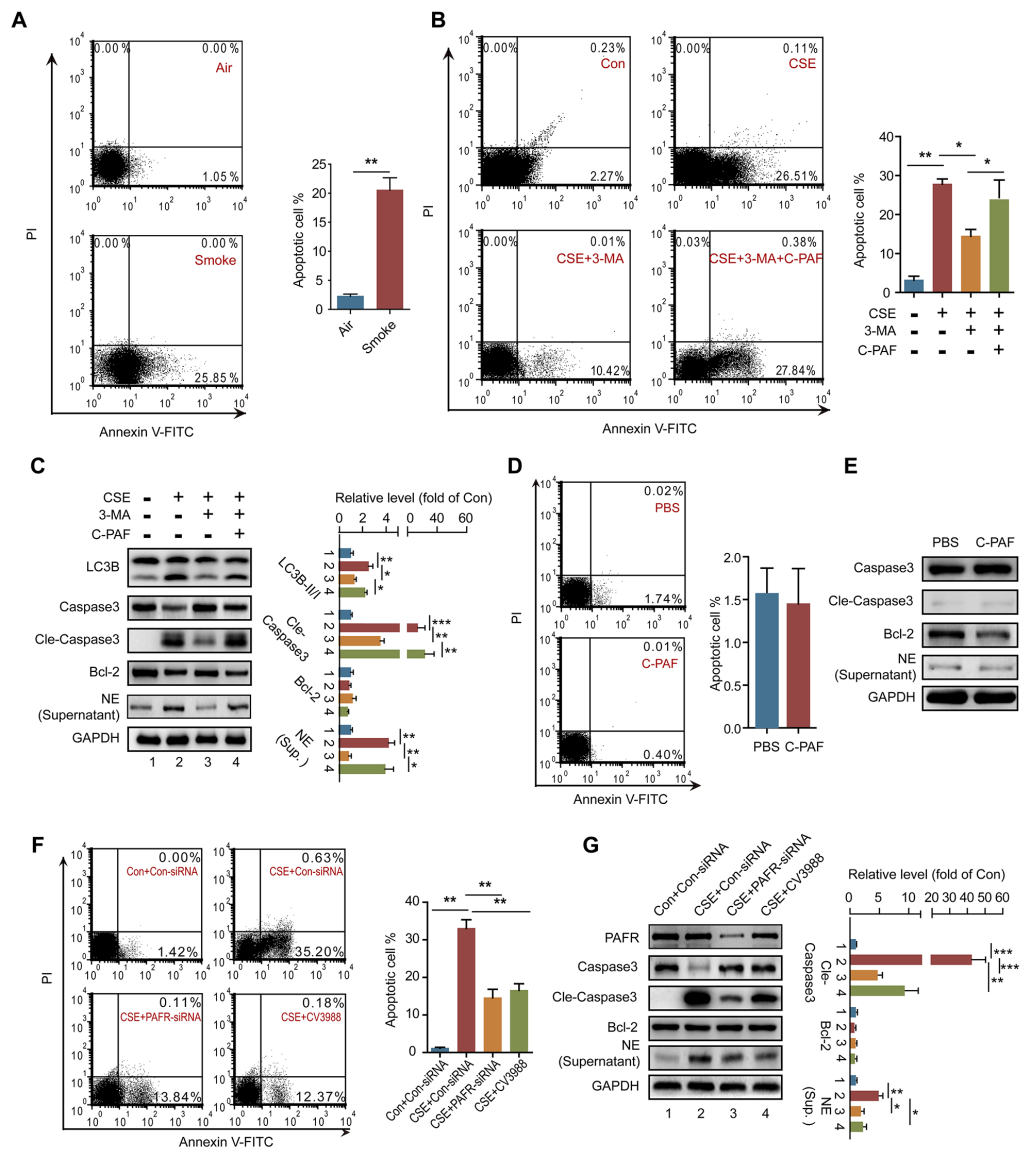
PAFR mediates CSE induced neutrophil death through autophagy **(A)** The number of apoptotic neutrophils in BALF from COPD mice was detected with flow cytometry (n = 6). **(B)** The number of apoptotic neutrophils from bone marrow (BM) was detected with flow cytometry after treatment with 5% CSE, 3-MA (10 mM), or/and C-PAF (5 μg/ml) for 24 hr (n = 3). **(C)** The expression of LC3B, apoptosis related proteins in BM neutrophils, and neutrophil elastase in the culture supernatant were detected with Western blot after treatment with 5% CSE, 3-MA (10 mM), or/and C-PAF (5 μg/ml) for 24 hr. Data are representative immunoblots and the ratios of the indicated protein to GAPDH (n = 3). **(D)** The number of apoptotic BM neutrophils was detected with flow cytometry after C-PAF (5 μg/ml) stimulation for 24 hr (n = 3). **(E)** The expression of apoptosis related proteins in BM neutrophils and neutrophil elastase in the culture supernatant were analyzed with Western blot after C-PAF (5 μg/ml) stimulation for 24 hr (n = 3). **(F)** The number of apoptotic BM neutrophils was detected with flow cytometry after 5% CSE exposure and indicated treatments for 24 hr (n = 3). **(G)** The expression of apoptosis related proteins in BM neutrophils and neutrophil elastase in the culture supernatant were detected with Western blot after CSE exposure and indicated treatments. Data are representative immunoblots and the ratios of the indicated protein to GAPDH (n = 3). Data are mean ± SEM. Statistical significance between two groups was determined by student t test; statistical significances among groups were determined by one-way ANOVA test; *P < 0.05, **P < 0.01, ***P < 0.001.

Neutrophil death is an important source for neutrophil-derived proteinases such as elastase and cathepsin G [33]. Indeed, the increased number of apoptotic neutrophils was observed in BALF obtained from CS-exposed mice in comparison with normal mice (Figure 4A). Similarly, CSE induced significant apoptosis of bone marrow neutrophils; but pharmacologic inhibition of autophagy by 3-MA prevented neutrophils from CSE triggered apoptosis (Figure 4B), which may result from the 3-MA inhibited autophagic core complex and autophagosome formation. In addition, 3-MA treatment reduced cleaved caspase 3, LC3B-II level, and inhibited elastase release from CSE treated neutrophils (Figure 4C). These data indicate that the over-activated autophagy results in autophagic death of neutrophils. Moreover, activation of PAFR abolished 3-MA-prohibited autophagic death of neutrophils and elastase release caused by CSE stimulation (Figure 4B and 4C). Indeed, directly activating PAFR by C-PAF neither induced neutrophil apoptosis nor promoted elastase release (Figure 4D and 4E). However, silencing or inhibiting PAFR prevented neturophils from autophagic death and reduced elastase release which respond to CSE treatment (Figure 4F and 4G). These data suggest that the activation of PAFR mediates the CSE-induced autophagic death of neutrophils during COPD development.

### PAFR induces autophagy by suppressing the Bcl-2/Beclin1 interaction

**Figure 5:**
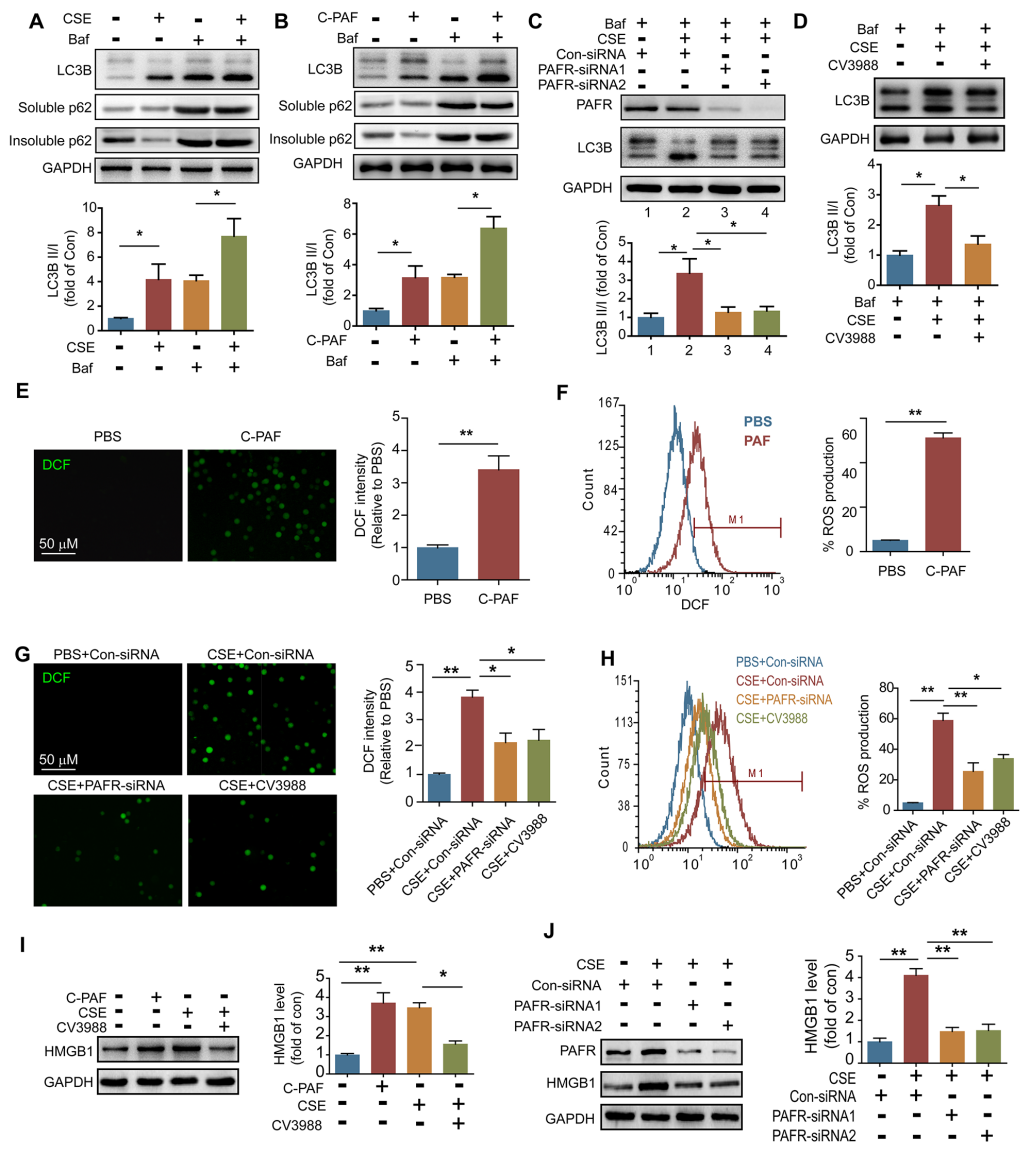
CS exposure increases ROS production in neutrophils **(A** and **B)** BM neutrophils were treated with 5% CSE (A), C-PAF (5 μg/ml) (B), or/and bafilomycin A1 (100 nM) for 12 hr, and the expression of LC3B and p62 was detected with Western blot. Data are representative immunoblots and the ratios of the indicated protein to GAPDH (n = 3). **(C)** LC3B level was detected with Western blot in PAFR-silenced BM neutrophils after indicated treatment (n = 3). **(D)** LC3B level was detected with Western blot in BM neutrophils after treatment with bafilomycin A1 (100 nM), 5% CSE, and CV3988 (30 μM) for 12 hr (n = 3). **(E** and **F)** BM neutrophils were treated with C-PAF (5 μg/ml) for 24 hr, and the ROS level was analyzed with immunofluorescence stain (E) and flow cytometry (F) (n = 3). **(G** and **H)** ROS production in BM neutrophils was evaluated with immunofluorescence stain (G) and flow cytometry (H) after indicated treatments. **(I)** The expression of HMGB1 was detected in BM neutrophils with Western blot after indicated treatments. Data are representative immunoblots and the ratios of the indicated protein to GAPDH (n = 3). **(J)** Con-siRNA or PAFR-siRNA transfected BM neutrophils were treated with 5% CSE for 24 hr, and the HMGB1 level was analyzed with Western blot. Data are representative immunoblots and the ratios of the indicated protein to GAPDH (n = 3). Data are mean ± SEM. Statistical significance between two groups was determined by student t test; statistical significances among groups were determined by one-way ANOVA test; *P < 0.05, **P < 0.01, ***P < 0.001.

**Figure 6:**
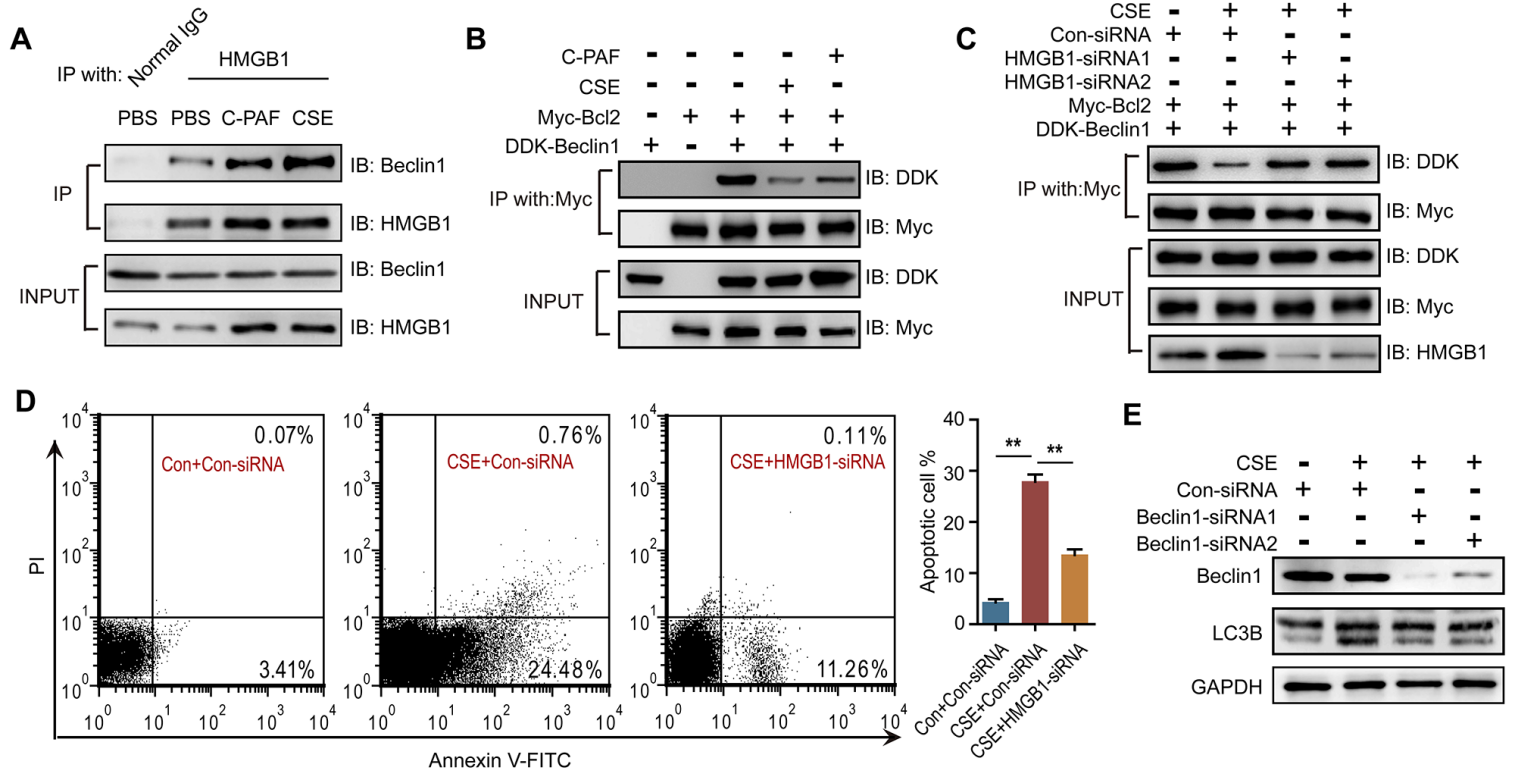
HMGB1 mediates PAFR induced autophagy by interfering with the Bcl-2/beclin1 interaction **(A)** IP assay analyses of the HMGB1/beclin1 interaction in BM neutrophils after 5% CSE or C-PAF (5 μg/ml) treatment for 24 hr (n = 3). **(B)** IP assay analyses of the Bcl-2/beclin1 interaction in BM neutrophils after indicated treatments (n = 3). **(C)** Con-siRNA or HMGB1-siRNA transfected BM neutrophils were treated with 5% CSE for 24 hr, and the Bcl-2/beclin1 interaction was detected with IP assay (n = 3). **(D)** The number of apoptotic neutrophils from BM was detected with flow cytometry after indicated treatment (n = 3). **(E)** BM neutrophils were transfected with Beclin-1-siRNA for 12 hr and then treated with 5% CSE for 24 hr, and the expression of LC3B was detected with Western blot. Data are mean ± SEM. Statistical significance between two groups was determined by student t test; statistical significances among groups were determined by one-way ANOVA test; *P < 0.05, **P < 0.01, ***P < 0.001.

To investigate the mechanism of PAFR-induced autophagy in CS-caused neutrophil death, we analyzed autophagic flux in CSE- and C-PAF- stimulated neutrophils using a LC3B-II/I ratio assay. CSE or C-PAF stimulation increased the level of LC3B-II, co-stimulation of the cells with CSE plus Bafilomycin A1 or C-PAF plus Bafilomycin A1 further increased LC3B-II, soluble and insoluble p62 levels in neutrophils, suggesting that both CSE and C-PAF caused LC3B-II accumulation is due to an enhanced formation of autophagosome (Figure 5A and 5B). Knockdown or inhibition of PAFR reduced the formation of autophagosome as indicated by a decreased LC3B-II/I turnover (Figure 5C and 5D). PAFR, a G-protein coupled receptor, positively contributes to inflammatory response, tumorigenesis and angiogenesis by enhancing the production of reactive oxygen species (ROS) [34, 35]. We verified that C-PAF or CSE treatment increased ROS level in neutrophils (Figure 5E-5H); silencing or inhibiting PAFR remarkably reduced ROS level in CSE treated neutrophils (Figure 5G and 5H). HMGB1 is an important nuclear protein which regulates the production of cytokines in neutrophils [36]; in addition, oxidative stress is a key regulator of HMGB1 production and releasing [37]. We observed that HMGB1 expression was enhanced in C-PAF or CSE treated neutrophils; however, knockdown or blocking PAFR attenuated CSE induced HMGB1 expression (Figure 5I and 5J). It has been reported that HMGB1 can activate autophagy by binding with beclin1 and disrupting the Bcl-2/beclin1 interaction [38]. We found that HMGB1 interacted with beclin1 in neutrophils and this interaction was increased after CSE or C-PAF treatment (Figure 6A). In parallel, the interaction between Bcl-2 and beclin1 was decreased in the presence of C-PAF or CSE (Figure 6B). Moreover, silencing HMGB1 recovered the association of Bcl-2 with beclin1 and reduced autophagic death in CSE-stimulated neutrophils (Figure 6C and 6D). To verify if the CSE induced autophagy is depend on beclin1, beclin 1 was silenced in CSE exposed neutrophils. We found that silencing beclin1 resulted in the inhibition of LC3B-II/LC3B-I conversion (Figure 6E). These finding illustrate that CSE induces autophagic death of neutrophils through increasing HMGB1 expression and promoting the formation of the autophagic core complex.

### Rupatadine attenuates COPD by inhibiting autophagic neutrophil death

**Figure 7:**
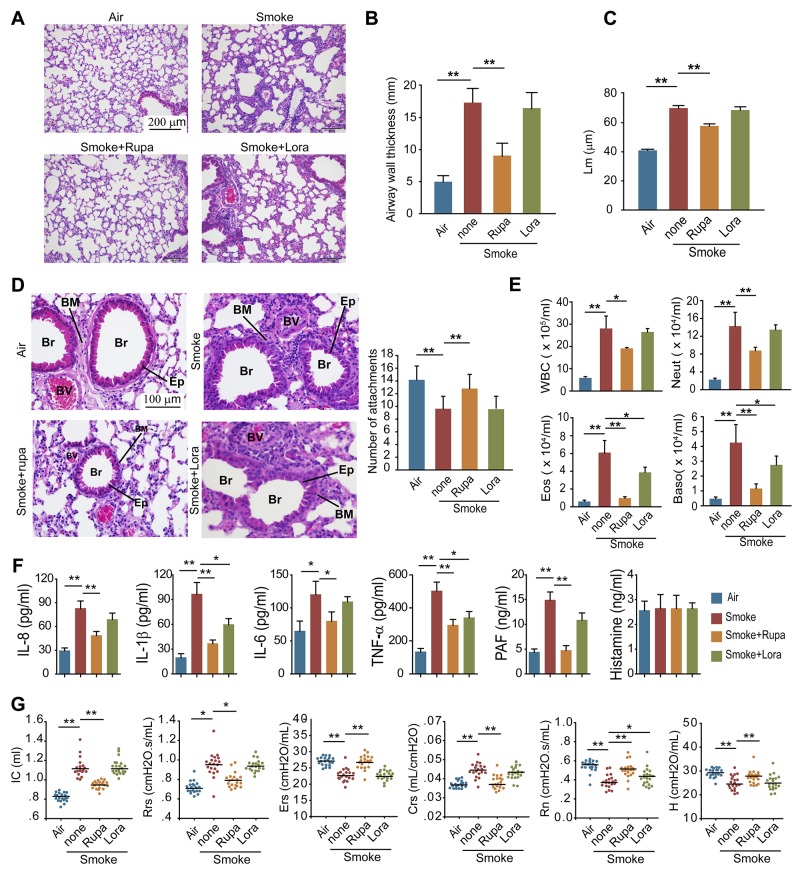
Rupatadine ameliorates CS induced COPD in mice **(A-D)** Mice were exposed with CS for 16 weeks, and were treated with rupatadine or loratadine (qd, ig) during the last 4 weeks of CS exposure. H&E-staining (A), airway wall thickness (B), the mean linear intercept of lung tissue (C), and number of alveolar attachments (D) were detected to evaluate the pathologic change of COPD in mice (Br: bronchia, BM: basement membrane, BV: blood vessel, Ep: epithelial cell) (n = 20). **(E)** The number of inflammatory cells was detected in BALF from the mice with the indicated treatment (n = 8). **(F)** The levels of IL-8, IL-1β, IL-6, TNF-α, PAF, and Histamine in BALF from the mice with the indicated treatment were evaluated with ELISA (n = 6). **(G)** Lung function of the mice was evaluated after 16 weeks CS exposure (n = 20). Data are mean ± SEM. Statistical significances among groups were determined by one-way ANOVA test; *P < 0.05, **P < 0.01, ***P < 0.001.

**Figure 8:**
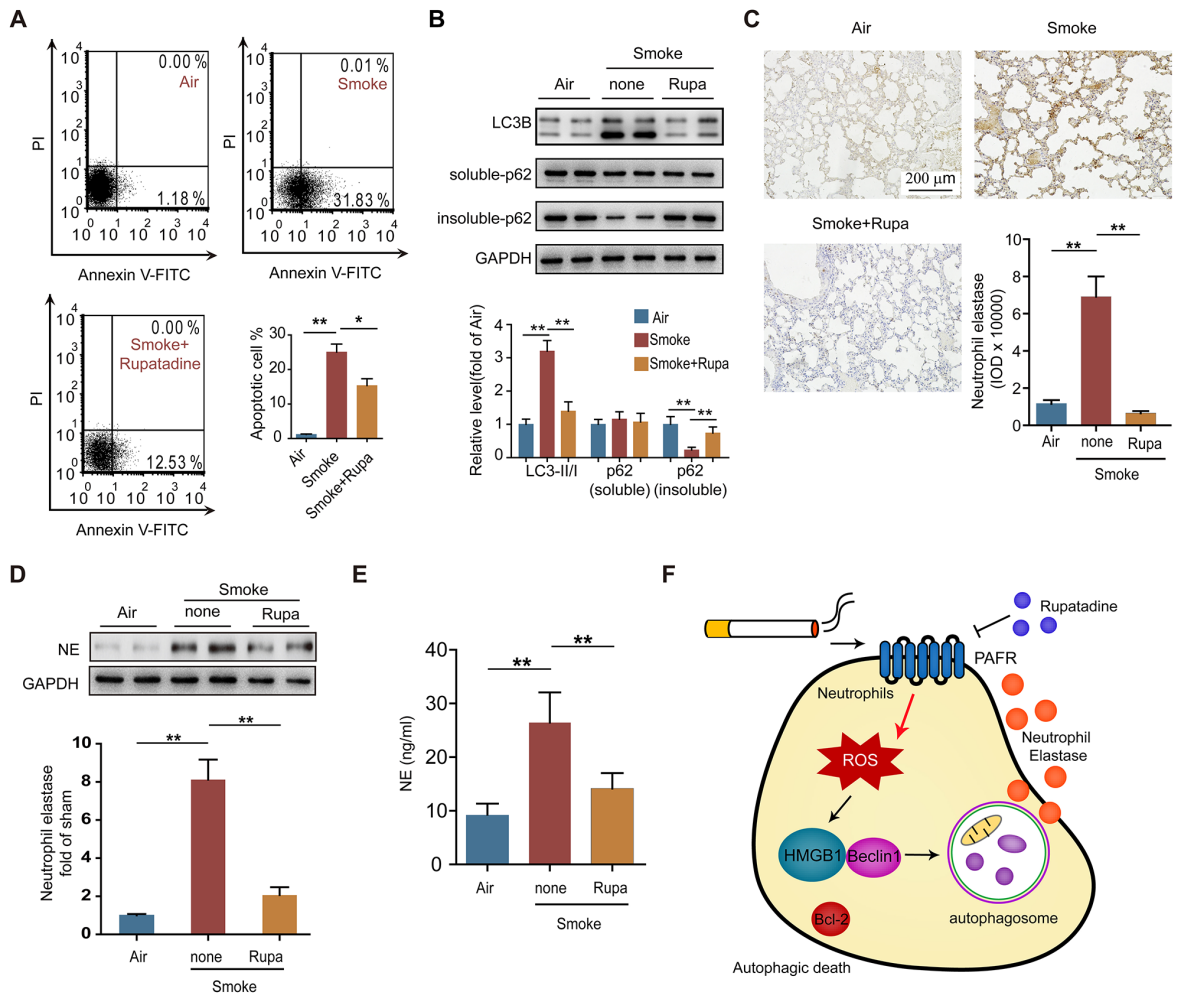
Rupatadine suppresses autophagic death of neutrophil in CS exposed mice **(A)** The number of apoptotic neutrophils in BALF from the indicated mice was evaluated with flow cytometry (n = 6). **(B)** Autophagy related proteins expression were detected with Western blot in lung tissue from the indicated mice. Data are representative immunoblots and the ratios of the indicated protein to GAPDH (n = 6). **(C** and **D)** The expression of neutrophil elastase in lungs from control and COPD mice was analyzed with immunohistochemistry (C) and Western blot (D) (n = 8). **(E)** The level of neutrophil elastase in BALF from control and COPD mice was evaluated with ELISA (n = 6). **(F)** Schematic diagram illustrates the role of PAFR-induced autophagy in COPD development. Data are mean ± SEM. Statistical significances among groups were determined by one-way ANOVA test; *P < 0.05, **P < 0.01, ***P < 0.001.

To verify whether PAFR could be a therapeutic target for the treatment of COPD, we observed the effects of rupatadine, a prescription dual antagonist for PAFR and histamine1 receptor (H1R), in mouse model of CS-induced COPD. H1R inhibitor loratadine was used as a control. Rupatadine, but not loratadine treatment significantly attenuated the enlargement of alveolar space (Figure 7A), reduced the airway wall thickness (Figure 7B) and mean linear intercept (Figure 7C), increased the number of alveolar attachments in the lung tissue from cigarette smoke exposed mice (Figure 7D). The number of white blood cells, neutrophils, eosinophils, and basophils were significantly reduced in BALF from rupatadine treated COPD mice; however, loratadine only decreased the counts of eosinophils and basophils (Figure 7E). Similarly, inhibiting PAFR by rupatadine significantly reduced the content of IL-8, IL-1β, TNF-α, and PAF in BALF from smoke exposed mice (Figure 7F). Lung function is a good standard for evaluating the progression of COPD both in clinic and pre-clinical studies. Rupatadine treatment reduced the inspiratory capacity, lung resistance, lung compliance, and increased lung elasticity, airway resistance (Rn), and tissue elasticity (H) of CS-exposed mice, suggesting rupatadine inhibits the small airway destruction and emphysema (Figure 7G). However, the improvement effect of loratadine on lung function was very limited (Figure 7G). As expected, the autophagic death ratio of neutrophils in BALF was decreased after inhibiting PAFR, which was indicated by Annexin V and PI staining, LC3B-II/I ratio, and the amount of insoluble p62 level (Figure 8A and 8B). In consistent with this finding, the NE levels in lung tissue and in BALF were reduced in CS-exposed mice treated with rupatadine (Figure 8C-8E). These results suggest that rupatadine is a promising medicine for the treatment of patients with COPD (Figure 8F).

## DISCUSSION

Cigarette smoke is the most important independent risk factor for the development of COPD as well as lung cancer and idiopathic pulmonary fibrosis. However, CS contains thousands of chemicals, which results in extremely complicated network of the mechanism involved in the pathogenesis of CS induced COPD. The present study illustrates that CS induced neutrophil death and elastase release are partly dependent on PAFR-medicated autophagy. The association of Bcl-2 and beclin1 was suppressed by HMGB1 whose expression was increased in neutrophil after CS stimulation. Inhibition of PAFR not only decreased HMGB1 level, suppressed autophagy activity, decreased NE release, but also produced therapeutic efficacy in mouse model of CS induced COPD.

Previous findings indicated that the content of PAF is increased in BALF and blood from COPD patients, and CS exposure promotes the synthesis of PAFR agonist in CS-exposed mice [39]. In addition, high level of PAFR is expressed on both large and small airway epithelium and alveolar epithelial cells in COPD patients [11, 12], which may facilitate PAFR-mediated inflammation. However, the mechanism whereby PAFR on airway epithelium promotes COPD development is unclear yet. *Streptococcus pneumoniae* is the most important bacteria during pathogenesis of COPD, which adherence to airway is mediated by PAFR [40], but whether this process contributes to airway obstruction is unknown. PAF is a potent biological molecule which contributes to the pathogenesis of many inflammatory diseases, such as allergy, asthma and pulmonary fibrosis [34, 41]. It has been reported that PAF can promote pro-survival activity and phagocytic capacity on neutrophils [14]. Moreover, PAF promotes the formation of neutrophil extracellular traps [15]. In this study, we found that C-PAF stimulation alone cannot induce neutrophil death but inhibiting PAFR activity blocks CSE triggered autophagic neutrophils death, suggesting that the specific chemical constituent of CS cooperates with PAFR-induced autophagy to promote COPD progression. In addition, these findings also indicate that CSE can activate PAFR on neutrophils, and this may result from autocrine PAF agonist induced by CSE exposure [42]. Thus, further investigation into the role of CS in activating PAFR is needed. In addition, we cannot rule out the possibilities of other G protein coupled receptors on neutrophils which were involved in CS induced COPD.

A number of studies indicate autophagy involved in the development of COPD. CS or CSE induces autophagy in human bronchial epithelial and in mouse airway cells by increasing the LC3B-II/I ratio [43, 44]. In addition, it has been proved that autophagy activity is increased in lung tissues from COPD patients [14], and LC3B^-/-^ mice shows resistance to CS induced emphysema and apoptosis [45]. However, the accumulations of p62 and ubiquitinated proteins were increased after the transiently activation of autophagy [46, 47], suggesting CS or CSE blocks autophagic flux in these epithelial cells and might cause airway inflammation [48]. It also has been reported that the autophagic flux status is depend on CSE concentration in epithelial cells [49]. Our results showed that the expression of beclin1 and the ratio of LC3B-II/I were increased in lung tissues form the mice after CS exposure. In parallel, beclin1 level was not enhanced in CS-exposed neutrophil, suggesting CS induces autophagy through various pathways in different types of cells. Furthermore, CSE could increase the LC3B-II level and promote AKT phosphorylation in neutrophil. These results illustrate that CSE induced autophagy in neutrophil is independent of the AKT/mTOR pathway. Indeed, we found that PAFR enhanced HMGB1 promotes the assembling of autophagy core complex by reducing the Bcl-2/beclin1 interaction in CSE treated neutrophils, suggesting that PAFR-HMGB1-Bcl-2-beclin1 pathway mediated the autophagy induction in CS exposed neutrophils, which contribute to the development of COPD.

Neutrophil is often considered as the effector cells in the pathogenesis of COPD through releasing proteinases such as elastase [9]. In addition, the inhibitor of NE is undergoing clinical trial for COPD therapy [50]. Previous studies suggested that reduced neutrophil apoptosis in circulation is a pathogenic factor in the development of COPD, and promoting neutrophil apoptosis has been considered as a therapeutic strategy for this respiratory disease [51]. In this study, we found that the number of dead neutrophils in BALF was increased from CS exposed mice, which is consistent with the previous report that increased apoptosis of neutrophils can be observed in sputum from COPD patients [10]. More importantly, some specific type of neutrophil death, such as NETosis could trigger the release of neutrophil extracellular traps (NETs), which are composed of elastase, cathepsin G, chromatin, and histone [28]. Interestingly, previous study confirmed that the formation of NETs requires autophagy activation [52]. The present study confirms that PAFR induced ROS production mediates autophagic neutrophil death after CSE treatment based on the following evidences: 1) autophagy was induced in CSE exposed neutrophils; 2) the induction of autophagy was parallel to neutrophils death; 3) blocking autophagy in neutrophils prevented CSE triggered cell death. Moreover, this process may eventually causes elastase release and lung injury. Thus, future studies should be directed at neutrophil apoptosis in the time course of COPD progression. Also, it has been reported that CSE can induce epithelial apoptosis either through autophagy or through Fas death receptor apoptotic pathway, indicating that CSE can trigger cell death through different mechanisms.

Despite recent mechanistic insights into the pathogenesis of COPD, therapeutics for this chronic diseases remain a significant clinical unmet need. COPD is already the third most common cause of death in world with the pathologic changes of progressive airway obstruction, emphysema, and fibrosis of small airways [1, 2]. Bronchodilator and the inhaled glucocorticoids are by far two of the most effective medicines to prevent COPD progression [53]. In general, these medicines cannot reverse COPD, but only control the symptoms during COPD progression. In this study, we found that PAFR antagonist rupatadine, which is a clinical used PAFR and H1 receptor dual inhibitor, suppresses CSE induced autophagic cell death, decreases the release of elastase from neutrophils, protects mice from CS caused COPD. However, using H1 receptor antagonist alone doesn’t show the therapeutic effects on COPD as rupatadine does in this study. These findings suggest that rupatadine may be a promising medicine for COPD therapy, especially in smoking patients. Moreover, the anti-inflammatory and anti-fibrosis features of rupatadine may also contribute to its anti-COPD effects [41, 54]. In summary, our current study indicates that PAFR is involved in the CS caused COPD through promoting autophagic neutrophil death and elastase release, which can be antagonized by rupatadine, a clinical used PAFR antagonist.

## MATERIALS AND METHODS

### Animal model of COPD

All animal procedures were conducted in accordance with the guidelines of ARRIVE and approved by the Institutional Animal Care and Use Committee of the Chinese Academy of Medical Sciences and Peking Union Medical College (Permit No. 002802). All protocols were in accordance with the approved guidelines. Male C57 BL/6 mice (18 g, 6-8 wk) (Vital River Lab Animal Technology Co., Ltd) were maintained in animal facility at the Institute of Materia Medica under Specific Pathogen Free (SPF) conditions. For animal studies, mice were earmarked before grouping and then were randomly separated into groups by an independent person. COPD mouse model was done according to previously published methods with modification [24–26]. Briefly, mice were exposed to cigarette smoke at a particle concentration of 200 mg/m^3^ in a chamber (60 ×35 ×30 cm, Shuang Yu, Beijing, China) for 4 hr a day, 5 days per week, for 16 weeks. Cigarette smoke exposure was performed using The University of Kentucky reference research cigarettes 3R4F (Lexington, KY, USA). Rupatadine (3 mg/kg) or Loratadine (3 mg/kg) was administered to the mice by oral gavage once a day during the last 4 weeks of CS exposure. 24 hr after the last administration, all the mice were anesthetized by avertin, and the lung function was measured. Lungs were excised and fixed for morphological evaluation or for measurement of protein expression.

### Reagent and antibody

Saponin, 3-MA and C-PAF (Carbamyl-PAF, a non-hydrolyzable bioactive analog of PAF) was purchased from Sigma-Aldrich (St. Louis, MO, USA). CV-3988 was purchased from ENZO life sciences (NY, USA). Bafilomycin A1 was purchased from Calbiochem (San Diego, CA, USA). Rupatadine was a generous gift from Zhejiang CiFu Pharmaceutics Inc. (Zhejiang, China). Loratadine was purchased from National institutes for Food and Drug Control (Beijing, China). Mouse neutrophil elastase, PAF and histamine ELISA kits were obtained from Cusabio Biotech Co., Ltd (Wuhan, China), whereas the other ELISA kits were purchased from eBioscience (San Diego, CA). Anti-mouse LC3B, neutrophil elastase antibodies were obtained from Abcam (Cambridge, UK); anti-mouse p62 antibody was purchased from Sigma- Aldrich. Anti-GAPDH antibody was purchased from Kangcheng Bio-tch (Shanghai, China). All the other antibodies were obtained from Cell Signaling technology.

### Lung function measurement

Mice were anesthetized with 400 mg/kg avertin by i.p injection and placed on a flexivent system (flexivent, SCIREQ Inc., Montreal, Canada). Then the mice were mechanically ventilated with a tidal volume of 10 ml/kg and respiratory rate of 150 breaths/min; 3 cmH2O positive end-expiratory pressure (PEEP) for evaluating lung function as described previously [41].

### Histological analysis

After the lung tissues were removed, the specimens were fixed with 4% paraformaldehyde then embedded in paraffin. Hematoxylin and eosin (H&E) staining was performed to determine the mean linear intercept. For detecting the airway wall thickness in the lung, all the sections were scanned at × 200 magnification, and the airway thickness was calculated using software Image-Pro plus 5.1.

### PAF-AH activity measurement

BALF was obtained from CS or air exposed mice, and the PAF-AH activity in BALF was detected using PAF acetylhydrolase inhibitor screening kit (BioVision).

### Neutrophils isolation and transfection

Neutrophils in BALF and bone marrow (BM) from mice were isolated using Neutrophil Isolation Kit (Miltenyi Biotec) according to manufacturer’s guideline. To obtain BALF, mice were sacrificed by over dosage avertin. The trachea was exposed using scissors. Using a 23 G needle to slowly inject 1 ml cold PBS with 0.1 mM EDTA into the lung, then collect the BALF from lungs. To obtain BM, femurs and shins were isolated with scissors from sacrificed mice, and then the bits of bone were removed. Flush the femurs and shins with 3 ml cold RPMI-1640 medium using a 25 G needle. The BM neutrophils were cultured in RPMI-1640 medium supplemented with 10% FBS and 1000 U/ml penicillin and 1000 μg/ml streptomycin. For plasmid transfection, the expression vector was transfected into BM neutrophils using the monocyte nucleofector kit (Lonza) according to the manufacturer’s instructions. Delivery of HMGB1-siRNAs (RIBOBIO) or PAFR-siRNAs (Santa Cruz) into cells was carried out with Lipofectamine RNA interference MAX Transfection Reagent (Life Technologies) following the manufacturer’s instructions.

### Immunoprecipitation

Co-immunoprecipitation experiments were per-formed as described previously [30]. Briefly, cells were collected and lysed for 30 min on ice. Soluble lysates were incubated with indicated antibodies at 4 °C overnight, followed by incubation with Protein A/G Plus–Agarose (Santa Cruz) at 4 °C for 2 hr. Immunocomplexes were separated from the beads and then boiled for 10 min. The precipitated proteins were subjected to SDS–PAGE and blotted with specific antibodies.

### Preparation of cigarette smoke extract

Cigarette smoke extract (CSE) was prepared as previous described [55] by bubbling single 3R4F cigarette smoke into 10 ml RPMI-1640 medium using a 50 ml plastic syringe. This conditional medium was filtered with a 0.22 μm filter and considered to be 100% CSE.

### Apoptosis assay for flow cytometry

Cells were stained with both FITC-Annexin V and PI (Beyotime). After 15 min incubation at 37 °C, stained samples were analyzed with flow cytometer (BD). For analysis apoptotic neutrophil in BALF, the cells were also stained with APC-Ly-6G antibody (Biolegend), and then the Ly-6G positive cells were detected with FITC-Annexin V and PI by flow cytometer.

### Autophagic assay

For detect autophagic flux, bone marrow neutrophils were infected with mRFP-GFP-LC3 adenoviral [30], and incubated with CSE or/and CV-3988 for 24 hr on a glass coverslips. For immunofluorescence staining, cells were fixed with 4% buffered paraformaldehyde for 15 min at room temperature. Images were acquired by confocal microscope (Leica Microsystems). For detect autophagosome formation, bone marrow neutrophils were transfected with GFP-LC3 plasmid, and stimulated with different concentration of CSE for 24 hr. Cells were harvested and washed with PBS containing 0.05% saponin. The GFP fluorescence was measured using flow cytometer.

### Western blot and immunostaining

Proteins were extracted from cells or lung tissues using RIPA buffer (Cell Signaling Technology), and the protein concentrations were determined with BCA Protein Assay Kit. The RIPA buffer-insoluble fraction was washed with PBS and resuspended in Laemmli SDS sample buffer. Protein sample were separated using SDS-PAGE, transferred to a PVDF membrane, and subjected to immunoblot analysis using specific antibody. The signaling was captured by a LAS4000 Image Station (General Electric Company, Fairfield, CT, USA). To evaluate the quantity of NE releasing from neutrophils, the culture supernatants were mixed with 5 × SDS loading, and the NE level was analyzed with immunoblot. The analysis of insoluble p62 was performed as previously report [30]. For immunohistochemical staining, the sections were scanned at × 200 magnification. The images were then digitalized, and the integrated optical density (IOD) of PAFR or NE were calculated using software Image-Pro plus 5.1.

### ROS measurement

Bone marrow neutrophils were treated with CSE (5%), C-PAF (5 μg/ml), or CV3988 (30 μM) for 24 hr, and intercellular ROS level was evaluated with dichlorofluorescin diacetate (DCFDA) (Abcam). Cells were stained with 20 μM DCFDA at 37 °C for 30 min. Then the cells were washed once, and the dichlorofluorescein (DCF) level was detected with confocal or flow cytometer.

### Statistics

Data are represented as the mean ± standard error of the mean (SEM). Student’s t test (2-tailed) was used to compare difference between two groups. One-way ANOVA with Tukey-Kramer’s comparison test was used to analyze difference among multiple groups. Generally, all assays were carried out with n ≥ 3 biological replicates. P < 0.05 was considered statistically significant.

## References

[1] Barnes PJ (2017). Senescence in COPD and its Comorbidities. Annu Rev Physiol.

[2] Anderson GP (2016). Advances in understanding COPD. F1000 Res.

[3] Kosmider B, Messier EM, Chu HW, Mason RJ (2011). Human alveolar epithelial cell injury induced by cigarette smoke. PLoS One.

[4] Holloway RA, Donnelly LE (2013). Immunopathogenesis of chronic obstructive pulmonary disease. Curr Opin Pulm Med.

[5] Barnes PJ (2016). Inflammatory mechanisms in patients with chronic obstructive pulmonary disease. J Allergy Clin Immunol.

[6] Blidberg K, Palmberg L, Dahlen B, Lantz AS, Larsson K (2012). Increased neutrophil migration in smokers with or without chronic obstructive pulmonary disease. Respirology.

[7] Costa C, Traves SL, Tudhope SJ, Fenwick PS, Belchamber KB, Russell RE, Barnes PJ, Donnelly LE (2016). Enhanced monocyte migration to CXCR3 and CCR5 chemokines in COPD. Eur Respir J.

[8] Pouwels SD, van Geffen WH, Jonker MR, Kerstjens HA, Nawijn MC, Heijink IH (2017). Increased neutrophil expression of pattern recognition receptors during COPD exacerbations. Respirology.

[9] Guyot N, Wartelle J, Malleret L, Todorov AA, Devouassoux G, Pacheco Y, Jenne DE, Belaaouaj A (2014). Unopposed cathepsin G, neutrophil elastase, and proteinase 3 cause severe lung damage and emphysema. Am J Pathol.

[10] Makris D, Vrekoussis T, Izoldi M, Alexandra K, Katerina D, Dimitris T, Michalis A, Tzortzaki E, Siafakas NM, Tzanakis N (2009). Increased apoptosis of neutrophils in induced sputum of COPD patients. Respir Med.

[11] Shukla SD, Muller HK, Latham R, Sohal SS, Walters EH (2016). Platelet-activating factor receptor (PAFr) is upregulated in small airways and alveoli of smokers and COPD patients. Respirology.

[12] Shukla SD, Sohal SS, Mahmood MQ, Reid D, Muller HK, Walters EH (2014). Airway epithelial platelet-activating factor receptor expression is markedly upregulated in chronic obstructive pulmonary disease. Int J Chron Obstruct Pulmon Dis.

[13] Sharma J, Young DM, Marentette JO, Rastogi P, Turk J, McHowat J (2012). Lung endothelial cell platelet-activating factor production and inflammatory cell adherence are increased in response to cigarette smoke component exposure. Am J Physiol Lung Cell Mol Physiol.

[14] Kuijpers TW, van den Berg JM, Tool AT, Roos D (2001). The impact of platelet-activating factor (PAF)-like mediators on the functional activity of neutrophils: anti-inflammatory effects of human PAF-acetylhydrolase. Clin Exp Immunol.

[15] Page C, Pitchford S (2013). Neutrophil and platelet complexes and their relevance to neutrophil recruitment and activation. Int Immunopharmacol.

[16] Tang D, Kang R, Coyne CB, Zeh HJ, Lotze MT (2012). PAMPs and DAMPs: signal 0s that spur autophagy and immunity. Immunol Rev.

[17] Shen HM, Codogno P (2011). Autophagic cell death: Loch Ness monster or endangered species?. Autophagy.

[18] Mizumura K, Cloonan SM, Haspel JA, Choi AM (2012). The emerging importance of autophagy in pulmonary diseases. Chest.

[19] Liu QP, Zhou DX, Lin P, Gao XL, Pan L, Jin FG (2013). Participation of autophagy in acute lung injury induced by seawater. Exp Lung Res.

[20] Mi S, Li Z, Yang HZ, Liu H, Wang JP, Ma YG, Wang XX, Liu HZ, Sun W, Hu ZW (2011). Blocking IL-17A promotes the resolution of pulmonary inflammation and fibrosis via TGF-beta1-dependent and -independent mechanisms. J Immunol.

[21] Chen ZH, Kim HP, Sciurba FC, Lee SJ, Feghali-Bostwick C, Stolz DB, Dhir R, Landreneau RJ, Schuchert MJ, Yousem SA, Nakahira K, Pilewski JM, Lee JS (2008). Egr-1 regulates autophagy in cigarette smoke-induced chronic obstructive pulmonary disease. PLoS One.

[22] Li D, Hu J, Wang T, Zhang X, Liu L, Wang H, Wu Y, Xu D, Wen F (2016). Silymarin attenuates cigarette smoke extract-induced inflammation via simultaneous inhibition of autophagy and ERK/p38 MAPK pathway in human bronchial epithelial cells. Sci Rep.

[23] Wang G, Zhou H, Strulovici-Barel Y, Al-Hijji M, Ou X, Salit J, Walters MS, Staudt MR, Kaner RJ, Crystal RG (2017). Role of OSGIN1 in mediating smoking-induced autophagy in the human airway epithelium. Autophagy.

[24] Givi ME, Peck MJ, Boon L, Mortaz E (2013). The role of dendritic cells in the pathogenesis of cigarette smoke-induced emphysema in mice. Eur J Pharmacol.

[25] Heulens N, Korf H, Cielen N, De Smidt E, Maes K, Gysemans C, Verbeken E, Gayan-Ramirez G, Mathieu C, Janssens W (2015). Vitamin D deficiency exacerbates COPD-like characteristics in the lungs of cigarette smoke-exposed mice. Respir Res.

[26] Jarnicki AG, Schilter H, Liu G, Wheeldon K, Essilfie AT, Foot JS, Yow TT, Jarolimek W, Hansbro PM (2016). The inhibitor of semicarbazide-sensitive amine oxidase, PXS-4728A, ameliorates key features of chronic obstructive pulmonary disease in a mouse model. Br J Pharmacol.

[27] Ostrovsky L, King AJ, Bond S, Mitchell D, Lorant DE, Zimmerman GA, Larsen R, Niu XF, Kubes P (1998). A juxtacrine mechanism for neutrophil adhesion on platelets involves platelet-activating factor and a selectin-dependent activation process. Blood.

[28] Concha C, Carretta MD, Alarcon P, Conejeros I, Gallardo D, Hidalgo AI, Tadich N, Caceres DD, Hidalgo MA, Burgos RA (2014). Oxidative response of neutrophils to platelet-activating factor is altered during acute ruminal acidosis induced by oligofructose in heifers. J Vet Sci.

[29] Hwang JW, Chung S, Sundar IK, Yao H, Arunachalam G, McBurney MW, Rahman I (2010). Cigarette smoke-induced autophagy is regulated by SIRT1-PARP-1-dependent mechanism: implication in pathogenesis of COPD. Arch Biochem Biophys.

[30] Hua F, Li K, Yu JJ, Lv XX, Yan J, Zhang XW, Sun W, Lin H, Shang S, Wang F, Cui B, Mu R, Huang B (2015). TRB3 links insulin/IGF to tumour promotion by interacting with p62 and impeding autophagic/proteasomal degradations. Nat Commun.

[31] Murrow L, Debnath J (2013). Autophagy as a stress-response and quality-control mechanism: implications for cell injury and human disease. Annu Rev Pathol.

[32] Klionsky DJ, Abdelmohsen K, Abe A, Abedin MJ, Abeliovich H, Acevedo Arozena A, Adachi H, Adams CM, Adams PD, Adeli K, Adhihetty PJ, Adler SG, Agam G (2016). Guidelines for the use and interpretation of assays for monitoring autophagy (3rd edition). Autophagy.

[33] Remijsen Q, Kuijpers TW, Wirawan E, Lippens S, Vandenabeele P (2011). Vanden Berghe T. Dying for a cause: NETosis, mechanisms behind an antimicrobial cell death modality. Cell Death Differ.

[34] Palgan K, Bartuzi Z (2015). Platelet activating factor in allergies. Int J Immunopathol Pharmacol.

[35] Anandi VL, Ashiq KA, Nitheesh K, Lahiri M (2016). Platelet-activating factor promotes motility in breast cancer cells and disrupts non-transformed breast acinar structures. Oncol Rep.

[36] Huebener P, Pradere JP, Hernandez C, Gwak GY, Caviglia JM, Mu X, Loike JD, Jenkins RE, Antoine DJ, Schwabe RF (2015). The HMGB1/RAGE axis triggers neutrophil-mediated injury amplification following necrosis. J Clin Invest.

[37] Min HJ, Kim JH, Yoo JE, Oh JH, Kim KS, Yoon JH, Kim CH (2017). ROS-dependent HMGB1 secretion upregulates IL-8 in upper airway epithelial cells under hypoxic condition. Mucosal Immunol.

[38] Tang D, Kang R, Livesey KM, Cheh CW, Farkas A, Loughran P, Hoppe G, Bianchi ME, Tracey KJ, Zeh HJ, Lotze MT (2010). Endogenous HMGB1 regulates autophagy. J Cell Biol.

[39] Sahu RP, Petrache I, Van Demark MJ, Rashid BM, Ocana JA, Tang Y, Yi Q, Turner MJ, Konger RL, Travers JB (2013). Cigarette smoke exposure inhibits contact hypersensitivity via the generation of platelet-activating factor agonists. J Immunol.

[40] Shukla SD, Fairbairn RL, Gell DA, Latham RD, Sohal SS, Walters EH, O'Toole RF (2016). An antagonist of the platelet-activating factor receptor inhibits adherence of both nontypeable Haemophilus influenzae and Streptococcus pneumoniae to cultured human bronchial epithelial cells exposed to cigarette smoke. Int J Chron Obstruct Pulmon Dis.

[41] Lv XX, Wang XX, Li K, Wang ZY, Li Z, Lv Q, Fu XM, Hu ZW (2013). Rupatadine protects against pulmonary fibrosis by attenuating PAF-mediated senescence in rodents. PLoS One.

[42] Makristathis A, Stauffer F, Feistauer SM, Georgopoulos A (1993). Bacteria induce release of platelet-activating factor (PAF) from polymorphonuclear neutrophil granulocytes: possible role for PAF in pathogenesis of experimentally induced bacterial pneumonia. Infect Immun.

[43] Zhou JS, Zhao Y, Zhou HB, Wang Y, Wu YF, Li ZY, Xuan NX, Zhang C, Hua W, Ying SM, Li W, Shen HH, Chen ZH (2016). Autophagy plays an essential role in cigarette smoke-induced expression of MUC5AC in airway epithelium. Am J Physiol Lung Cell Mol Physiol.

[44] Furlong HC, Stampfli MR, Gannon AM, Foster WG (2015). Cigarette smoke exposure triggers the autophagic cascade via activation of the AMPK pathway in mice. Biol Reprod.

[45] Chen ZH, Lam HC, Jin Y, Kim HP, Cao J, Lee SJ, Ifedigbo E, Parameswaran H, Ryter SW, Choi AM (2010). Autophagy protein microtubule-associated protein 1 light chain-3B (LC3B) activates extrinsic apoptosis during cigarette smoke-induced emphysema. Proc Natl Acad Sci USA.

[46] Vij N, Chandramani P, Westphal CV, Hole R, Bodas M

[47] Tran I, Ji C, Ni I, Min T, Tang D, Vij N (2015). Role of Cigarette Smoke-Induced Aggresome Formation in Chronic Obstructive Pulmonary Disease-Emphysema Pathogenesis. Am J Respir Cell Mol Biol.

[48] Gu W, Cui R, Ding T, Li X, Peng J, Xu W, Han F, Guo X (2017). Simvastatin alleviates airway inflammation and remodelling through up-regulation of autophagy in mouse models of asthma. Respirology.

[49] Mercado N, Colley T, Ito K, Barnes P (2014). Cigarette smoke impairs autophagic flux in bronchial epithelial cells. Eur Respir J.

[50] Kuna P, Jenkins M, O'Brien CD, Fahy WA (2012). AZD9668, a neutrophil elastase inhibitor, plus ongoing budesonide/formoterol in patients with COPD. Respir Med.

[51] Zhang J, He J, Xia J, Chen Z, Chen X (2012). Delayed apoptosis by neutrophils from COPD patients is associated with altered Bak, Bcl-xl, and Mcl-1 mRNA expression. Diagn Pathol.

[52] Remijsen Q, Vanden Berghe T, Wirawan E, Asselbergh B, Parthoens E, De Rycke R, Noppen S, Delforge M, Willems J, Vandenabeele P (2011). Neutrophil extracellular trap cell death requires both autophagy and superoxide generation. Cell Res.

[53] Vanfleteren LE, Spruit MA, Wouters EF, Franssen FM (2016). Management of chronic obstructive pulmonary disease beyond the lungs. Lancet Respir Med.

[54] Alevizos M, Karagkouni A, Vasiadi M, Sismanopoulos N, Makris M, Kalogeromitros D, Theoharides TC (2013). Rupatadine inhibits inflammatory mediator release from human laboratory of allergic diseases 2 cultured mast cells stimulated by platelet-activating factor. Ann Allergy Asthma Immunol.

[55] Xu Y, Li H, Bajrami B, Kwak H, Cao S, Liu P, Zhou J, Zhou Y, Zhu H, Ye K, Luo HR (2013). Cigarette smoke (CS) and nicotine delay neutrophil spontaneous death via suppressing production of diphosphoinositol pentakisphosphate. Proc Natl Acad Sci USA.

